# Bioengineered Wound Healing Skin Models: The Role of Immune Response and Endogenous ECM to Fully Replicate the Dynamic of Scar Tissue Formation In Vitro

**DOI:** 10.3390/bioengineering9060233

**Published:** 2022-05-27

**Authors:** Francesco Urciuolo, Roberta Passariello, Giorgia Imparato, Costantino Casale, Paolo Antonio Netti

**Affiliations:** 1 Interdisciplinary Research Centre on Biomaterials (CRIB), University of Naples Federico II, P.le Tecchio 80, 80125 Naples, Italy; costantino.casale@unina.it (C.C.); paoloantonio.netti@unina.it (P.A.N.); 2 Department of Chemical, Materials and Industrial Production Engineering (DICMAPI), University of Naples Federico II, P.le Tecchio 80, 80125 Naples, Italy; roberta.passariello@unina.it; 3 Center for Advanced Biomaterials for HealthCare@CRIB Istituto Italiano di Tecnologia, Largo Barsanti e Matteucci 53, 80125 Naples, Italy; giorgia.imparato@iit.it

**Keywords:** scar tissue, engineered skin, skin on chip, wound healing, immune response, inflammation

## Abstract

The healing of deep skin wounds is a complex phenomenon evolving according with a fine spatiotemporal regulation of different biological events (hemostasis, inflammation, proliferation, remodeling). Due to the spontaneous evolution of damaged human dermis toward a fibrotic scar, the treatment of deep wounds still represents a clinical concern. Bioengineered full-thickness skin models may play a crucial role in this direction by providing a deep understanding of the process that leads to the formation of fibrotic scars. This will allow (i) to identify new drugs and targets/biomarkers, (ii) to test new therapeutic approaches, and (iii) to develop more accurate in silico models, with the final aim to guide the closure process toward a scar-free closure and, in a more general sense, (iv) to understand the mechanisms involved in the intrinsic and extrinsic aging of the skin. In this work, the complex dynamic of events underlaying the closure of deep skin wound is presented and the engineered models that aim at replicating such complex phenomenon are reviewed. Despite the complexity of the cellular and extracellular events occurring during the skin wound healing the gold standard assay used to replicate such a process is still represented by planar in vitro models that have been largely used to identify the key factors regulating the involved cellular processes. However, the lack of the main constituents of the extracellular matrix (ECM) makes these over-simplistic 2D models unable to predict the complexity of the closure process. Three-dimensional bioengineered models, which aim at recreating the closure dynamics of the human dermis by using exogenous biomaterials, have been developed to fill such a gap. Although interesting mechanistic effects have been figured out, the effect of the inflammatory response on the ECM remodelling is not replicated yet. We discuss how more faithful wound healing models can be obtained by creating immunocompetent 3D dermis models featuring an endogenous ECM.

## 1. Introduction

The increasing number of traumas and pathologies make skin wounds a major healthcare problem due to the impaired regeneration process occurring during the healing phase. Healing of deep skin wounds involves both complex interactions between different cell types and a fine spatiotemporal regulation of different biological phenomena (hemostasis, inflammation, proliferation, remodeling) [1]. In superficial lesions, the damaged epidermis is able to regenerate forming a neo-tissue that is indistinguishable from the undamaged one. On the contrary, the dermis heals through a repair process that leads to the formation of the scar [2,3,4]. Although complex, the repair process is a “mere” closing of the gap with the final aim of restoring the mechanical integrity of the damaged area, preventing the loss of blood and body fluids and pathogens invasion. The repair is characterized by an inflammatory process that leads to an impaired build-up of the ECM of the dermis, resulting in a dysfunctional tissue and presenting: (i) a high rate of differentiation of fibroblasts into myofibroblast lineage (ii) increased collagen content and stiffness, (iii) severe contractures, (vi) loss of sensitivity, and (v) loss of vascularization. Then, the abnormal three-dimensional (3D) microenvironment hinders the morphogenesis of skin appendages (i.e., hair follicles and glands) [5,6,7]. Finally, the dysfunctional dermis-epidermis interface formed in the repaired skin results in altered skin permeability, compromising both the trafficking of substances and the thermoregulation capability [8]. This set of events, taken together, can seriously compromise the quality of life of patients affected by extended and deep wounds. Apart from aesthetic concerns, the impaired dermis remodeling generates high social and human costs in terms, for example, of expensive therapies and treatments, prolonged hospitalizations, rehabilitations, and social discomforts. Understanding the key events involved in tissue repair is still a challenge and may result crucial for the development of strategies that can guide human cells toward a regenerative process rather than a repairing one. Due to the complexity of the wound healing process, to date, animal models and humans remain the most commonly used platform to study the mechanism of scar formation [9,10,11,12,13]. Nevertheless, the former are not predictive of the human pathophysiologic state and dynamics [14], while the latter are limited by scarce availability and ethical concerns. Against this background, tissue engineering has tried to recapitulate the mechanisms of wound closure in vitro by designing platforms that mimic the human skin, or its parts, able to undergo the closure process when damaged.

In the present review, after outlining the most relevant events that occur during wound closure, we analyze 2D and 3D tissue models used to replicate in vitro wound healing phases. This biological process and the consequent formation of scar tissue are phenomena that occur mainly at the level of the dermis and the ECM. Therefore, we highlight that in vitro 3D models should represent more reliable and truthful systems in reproducing such mechanisms. Nevertheless, we discuss how the 3D models based on exogenous matrices fail in replicating the modifications of the ECM during the closure process. For this reason, we argue that bioengineered human dermis in which fibroblasts are continuously engaged in the production of their own ECM (endogenous 3D models), due to their capability in replicating the ECM modifications occurring in the native dermis during the closure. The importance of endogenous ECM has also been discussed for in vivo applications as a new tissue engineering tool for completely restoring skin functions after serious injuries [15].

Finally, the coupling of such responsive 3D bioengineered human dermis with immune and inflammatory response, is suggested as an improvement of the models for the replication of the scar tissue formation in vitro.

## 2. The Wound Healing Process

**Figure 1:**
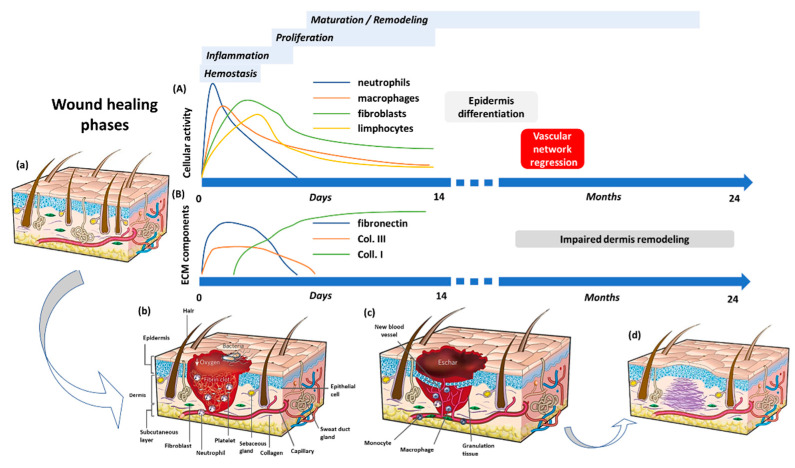
Schematic representation of wound healing phases: (**A**,**B**) Temporal evolution of cell-mediated wound healing and relative ECM components synthesis, (**a**) Undamaged skin, (**b**) Hemostatic phase and immune cells invasion in the wound bed, (**c**) Proliferation phase characterized by keratinocytes, fibroblasts and endothelial cells migration and proliferation for angiogenesis, collagen deposition, granulation tissue formation, re-epithelialization, and wound contraction to occur, (**d**) Maturation phase consisting in collagen remodeling (from type III to type I) and wound closure. Reprinted from Gurtner GC, Werner S, Barrandon Y et al. Wound repair and regeneration. Nature 2008; 453:314–21 [16] with permission from Springer Nature.

Wound healing is a complex mechanism that spans over different temporal and spatial scales and aims to restore the architecture, morphology, and functionality of the injured tissue. Starting from the regeneration of residual undamaged cells, the healing cascade will end up in the deposition of new connective tissue that eventually becomes a scar. To accomplish this task, the precise recruitment and coordination of different cell populations are of paramount importance, occurring through mechanical and biochemical regulatory networks during four distinct but overlapping phases (Figure 1).

Upon injury, the tissue barrier properties are lost, thus initiating the finely tuned and controlled cascade of wound healing stages regulated by a sequence of biologically active species (grow factors, cytokines, chemokines) [17]. These factors activate the different cell types and are released by the cells themselves to produce effects that are essential for the correct restoration of the complex three-dimensional architecture of any tissue (Table 1). This biological process begins with the hemostatic phase in which blood and lymphatic vessels undergo vasoconstriction to reduce blood and lymph loss. In addition, a new fibrin cap is formed, providing an early wound coverage and a simple scaffold for subsequent cell infiltration and migration. As the wound extends to the endothelium, circulating platelets are activated thanks to the adhesion to the underlying collagen fibers. Once activated, they begin to secrete their cytoplasmic granules content: coagulation factors that form the intrinsic and extrinsic pathways leading to a stable fibrin clot development, and a plethora of growth factors that enhance the chemotaxis and the division of the cells which are part of the other stages of wound healing [16,18,19,20]. The first to respond to the platelets’-releasing chemotactic factors call are immune system cells that mount an inflammatory response as soon as they penetrate inside the wound site. This second phase, characterized by the presence of erythema, heat, oedema, and pain, has the main objective of preventing infection and cleaning up the wound thanks to the destruction of any pathogens as well as cellular and matrix debris. As a fundamental part of innate immunity, neutrophils are the first to appear and are mainly responsible for invasion management and phagocytosis of microorganisms, capturing them through neutrophil extracellular traps (NETs) and destroying them through the secretion of reactive oxygen species (ROS) and antimicrobial peptides and proteases. Apoptotic neutrophils, additionally, produce important chemotactic cytokines and growth factors, such as IL-17 and vascular endothelial growth factor (VEGF), thus allowing the entrance of monocytes from the blood stream, that will later differentiate into macrophages [21]. In wound healing investigations, specifically, those concerning the role of the inflammatory response, both monocyte-derived and tissue-resident macrophages have been widely studied since they are considered to be the key players in this complex repair mechanism. In response to microenvironmental signals, macrophages change their phenotype, making their presence crucial: they switch from a pro-inflammatory (M1) to an anti-inflammatory (M2) state mediating the entire wound healing process. Soon after the injury, the recruited macrophages switch to the M1 configuration to create a more inflammatory, pathogen-killing environment by releasing pro-inflammatory molecules such as interferon-γ (IFN-γ), IL-1, IL-6, IL-12, IL-23, and tumor necrosis factor α (TNFα). In addition, they remove cell fragments, degrade the broken matrix by synthesizing several matrix metalloproteinase (MMP) and digest neutrophils to avoid non-specific tissue degradation and incessant inflammation. In the last part, factors such as fibroblast growth factor-2 (FGF-2), platelet-derived growth factor (PDGF), and VEGF are released, which are responsible for more immune cells to settle in and promote the proliferation of endothelial cells, fibroblasts, and epithelial cells. At this stage, a new provisional tissue begins to form inducing an alternative activation of macrophages, the M2 phenotype. Anti-inflammatory macrophages with the help of transforming growth factor-β1 (TGF-β1), VEGF, FGF, and PDGF support inflammation suppression and drive new ECM synthesis, wound contraction, and angiogenesis [22,23]. Endless studies attempt to find an explanation for the presence of T lymphocytes even when no suspicious contamination is present [21,23,24]. Their role in wound healing is still defined as unknown or irrelevant but recent studies have shown that they are important for tissue remodeling and infection resolution. As with macrophages, it has been seen that the TH1/TH2 paradigm and, therefore, the dual nature of this cell type, contributes both to the formation of a phlogistic environment and the replacement of injured tissue with new tissue [25]. Regarding the first aspect, IFN-γ produced by M1 macrophages controls CD4+ type 1 T helper cells (TH1) polarization, which promotes the preliminary pro-inflammatory wound microenvironment. As with the M2 phenotype, there is a specific subset of CD4+ T cells, TH2 cells, that have the potential to shift the overall balance of inflammatory factors in a wound towards a more regenerative or fibrotic outcome. This type of helper T cells is primarily activated by molecules released from dying cells due to damage, known as damage-associated molecular patterns (DAMPS), and contributes to the final stages of wound healing through the release of IL-4 and IL- 13, which support the proliferation of fibroblasts and the deposition of ECM [26]. Once the inflammatory phase is over, healing continues with the final two phases, which involve the formation and remodeling of a new ECM. These stages are largely dependent on the anti-inflammatory cells of the immune system (M2 macrophages and TH2 cells) as a prominent source of cytokines (IL-4, IL-13) and growth factors (TGF-β1) that promote matrix deposition and differentiation of fibroblasts into myofibroblasts, angiogenesis, and re-epithelialization. The proliferative phase, as the name suggests, involves the recruitment and proliferation of the cells necessary for the formation of new tissue. As a platform for the adhesion and movement of fibroblasts, endothelial and epithelial cells, the granulation tissue, so-called because of its high cellular content, is preliminarily reproduced. This new tissue is generated by the synthesis and deposition of the components of the ECM by fibroblasts. Also, in this case, both the fibroblasts deriving from the differentiation of fibrocytes, and undamaged fibroblasts present in the tissue migrate and proliferate in the injured tissue and are positioned in layered planes, parallel to the surface of the epithelium. In the same configuration, type I and type III collagen fibers are released, allowing the matrix to be easily contracted, thus reducing the exposed wound volume. Subsequently, the fibroblasts differentiate into myofibroblasts, which can be recognized by the expression of alpha-smooth muscle actin (α-SMA), and cause the contraction of the wound by grasping the flaps of the same [21,27]. Once a stroma equivalent has formed, it requires the supply of oxygen and nutrients hence new blood vessels need to be formed. In response to hypoxia and high lactic acid concentration, platelets and macrophages release growth factors, such as VEGF, which stimulate endothelial cells to proliferate and form new capillaries [21,28]. Lastly, the wound completely closes as a result of the re-epithelialization process carried out by the epithelial cells, present at the edges of the lesion. In the presence of epidermal growth factor (EGF) released by platelets and transforming growth factor- α (TGF-α) released by both macrophages and platelets, a phenotypic change occurs. This leads to the abandonment of desmosomes and hemidesmosomes and to the expression of actin filaments that allow cell detachment and subsequent migration. This process is facilitated by plasmin which removes the fibrin clot, and collagenase, which digests damaged stroma [19,29]. At this point, the granulation tissue enters the remodeling phase, which can take years to reach equilibrium. Initially, the development of new tissue stops with the apoptosis of cells that are no longer needed, as well as the pruning of non-functional vessels. Moreover, overly produced ECM components get degraded by the action of collagenase and MMPs secreted by fibroblasts, macrophages, endothelial and epithelial cells. During tissue maturation, type III collagen is completely replaced by type I collagen; the collagen fibers align along tension lines and water is reabsorbed to facilitate filaments approach and cross-link. This type of collagen is aligned in small parallel bundles and is, therefore, different from collagen that is randomly arranged in healthy tissue [19,21]. In humans, due to the lack of regenerative capacity, this process can end with the formation of scar tissue that cannot replicate neither the function or the structure of the normal tissue. In particular, scar differs from healthy tissue in terms of composition, mechanical properties, color, vascularity, and formation of the dermal, epithelial, and vascular adnexa. This is largely due to the rapid intrinsic nature of the reparative response at the expense of perfect but slower morphological and functional regeneration. Additionally, the scar can only replicate 80% of the tensile strength of healthy tissue. This phenomenon, however, does not occur in a fetal tissue which instead follows a predominantly regenerative path. Healing dynamics studies of fetal tissue have shown how the failure to assemble an adequate inflammatory response, due to the absence of immune cells and, therefore, also of the factors that normally secrete, results in perfectly regenerated tissue, capable of forming the intricate dermal and epidermal adnexa and scarless [30]. The only human compartment that resembles this behavior is the oral mucosa. The wounds of the oral mucosa are characterized by an extremely reduced secretion of pro-inflammatory cytokines and this generates an unfavorable environment for the infiltration of the immune system cells. The main consequence of this phenomenon is the reduction of fibroblasts activity and, consequently, a non-excessive deposition of collagen fibers [31]. As described in this section, the inflammatory response plays a central role in the coordination of the different healing phases. In particular, macrophages and T cells make a huge contribution both in the initial part to prevent the onset of infection and in the final part by maneuvering the steps that lead to the generation of new tissue. For this reason, a thorough characterization is needed, making use of a 3D model that can better recapitulate the complex architecture found in vivo. Indeed, tissue damage is something that concerns the extracellular components and their assembly, not just the cellular aspect. Tissue remodelling, and scarring are biological processes, triggered by trauma and inflammation, occurring at the ECM level. This must first be reconstituted and, subsequently, regain its role as a modulator of cell-cell and cell-matrix communication to ensure that the repair algorithm proceeds in a controlled and well-organized manner.

## 3. 2D Models: An over Simplified View of the Repair Dynamics

**Figure 2:**
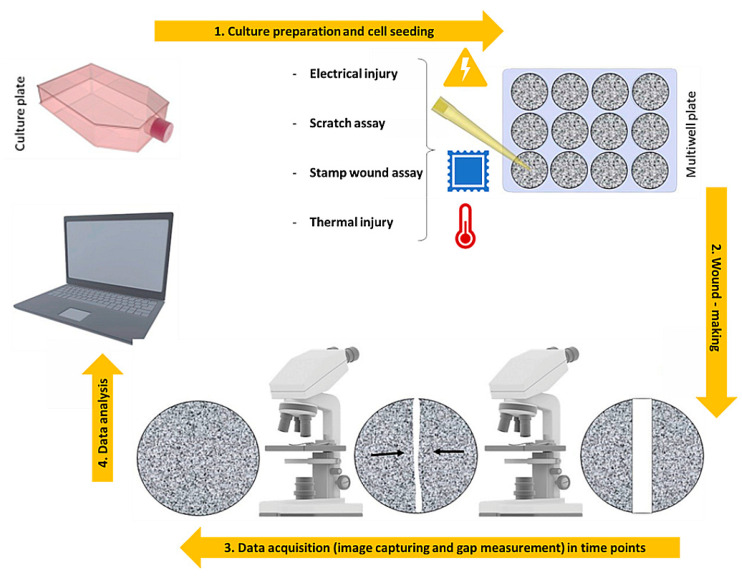
2D scratch-assay steps: (**1**) Cell culture preparation and cell seeding into a plastic-bottomed multiwell tissue culture plates, (**2**) Cells confluent monolayer scratching with a pipette tip, (**3**) Time-lapse brightfield image acquisition of wounds up to gap closure, (**4**) Data analysis for mean cells migration rate estimation. Readapted from Grada A, Otero-Vinas M, Prieto-Castrillo F et al. Research Techniques Made Simple: Analysis of Collective Cell Migration Using the Wound Healing Assay. J Invest Dermatol 2017; 137:e11–6 [33] with permission from Elsevier.

Numerous models, both in vitro and in vivo, have been developed to study the complex dynamics of wound healing and to perform various tests of new therapeutic strategies to manipulate tissue repair, including animal models, 2D cell cultures, and 3D models. Two-dimensional in vitro models of wound healing are primarily designed as simple and inexpensive tests consisting of four steps (Figure 2) to model and study cell movement in a controlled in vitro environment [32,33].

First, a monolayer of confluent cells, representing the in vivo conditions of the tissue before wounding is cultured on a specific substrate. Usually, cell types suitable for in-sheet migration [34], such as epithelial and endothelial cells, are used to generate the monolayer for this type of analysis. After reaching confluency, a cell-free space mimicking the lesion is created by the use of mechanical, thermal, electrical, or optimal means (Table 2). The healing phase of the resulting wound is monitored by taking time-lapse images and videos with a simple optical microscope or by using fluorescence to assess the expression of specific proteins and molecules produced. Additionally, staining the nuclei of cells provides indications of their behavior. These procedures are also used to measure the distance between the edges of the wound or the rate of change in covered area. One of the earliest and still most used mechanical methods is the scratch assay, which involves scraping the cell mat into a straight line and measuring the rate at which these cells migrate to fill the gap. The wide application is due to its very simple protocol and to the implementation of scratching devices that are easily available in the laboratory, such as a pipette tip [35], cell scrapers [36,37], toothpicks [38], and metallic micro indenters [38]. Another possible mechanical method consists of the disintegration of the cell monolayer by applying a pressure force through a mold, usually made of rubber [39] or polydimethylsiloxane (PDMS) [40]. The innovation of this technique relies on the possibility of reproducing lesions of different shapes and geometries, quite precisely, by simply varying the mold used [39,40]. Although easy, fast, and cheap, one of the major drawbacks is the manual execution of the injury, which reduces the repeatability of the experiment. Manual performance can lead to the formation of irregular gaps and lesions at different positions, and the likely destruction of ECM components used as coatings on the culture plates may also occur [41]. In addition, this type of technique may affect the subsequent migration and proliferation phases, as the debris from damaged cells cannot be easily removed, thus accumulating in the empty area. Consequently, the consistency of data acquisition and processing will be invalidated [42]. To limit these downsides, attempts have been made to automate the process through mechanical devices that make the damage operator-independent and achieved in seconds. These include the WoundMaker™, a 96-pin mechanical device designed to create homogeneous, 700–800 µm wide wounds in cell monolayers on 96-well microplates [43], or the use of pipettes controlled from a workstation [44,45]. A further development of these techniques is thermal damage combined with mechanical damage, since it is the closest realistic approximation to the most common form of injury to which the skin is normally subjected, such as accidental contact with hot or excessively cold uncovered objects. This type of damage can be reproduced, for example, by using thermally controlled molds [46]. Because it is a thermomechanical application, it shares the disadvantages of purely mechanical injury techniques combined with a transfer of heat toward the cells at the wound border. This results in an increase of the injury, leading to non-reproducible lesion sizes. Alternative techniques to thermomechanical injury are those which use electrical or optical wound sources. In the electrical injury assay, the cells are cultured on small gold electrodes [47,48,49] deposited on the bottom of tissue culture platforms, while a larger counter electrode is inserted into the culture medium, and used as an electrolyte. Such devices have been widely used by applying a weak alternative current (AC) (<1 uA) that causes a voltage drop of a few millivolts, resulting in non-invasive effects on the cells but simply changes in the electrical impedance of the system. Impedance change monitoring has been exploited to study various cell dynamics such as cell growth, barrier properties, or the effect of introducing particular compounds. One of the advantages of this automatic electrical injury method is the production of repeatable gaps of a precise diameter of 250 µm that heal within a few hours, compared to its mechanical counterpart which is inaccurate and takes at least 1 day. Additionally, thanks to the insulating properties of the cell membrane, which lead to a drastic increase in electrical impedance, the dynamics of wound healing, from destruction to regrowth, can be studied in real time, simply by measuring this electrical parameter rather than using classical microscopy. Nevertheless, there are some negative notes here as well. Firstly, the cell layer will anchor firmly to the electrodes, making it difficult to detach. Moreover, this technique follows a very precise protocol and any variation in terms of cell adhesion and density will inevitably lead to altered electrical impedance measurements. Currently, the disadvantage that deserves more attention is the heat source generated by the high current intensity during lesioning, which also leads to the destruction of neighboring cells close to those grown on the electrodes. Finally, specialized and expensive instrumentation is required for this type of experiment. Another method involves the use of optical abrasion techniques which have been developed with instruments that exploit a laser beam used both to create a wound on the cell monolayer and to record cell migration and wound closure through bright-field imaging [50]. Zordan′s group [50] has demonstrated that this optical methodology yields highly reproducible and high-throughput results by controlling the size and shape of the lesion with the Laser Enabled Analysis and Processing instrument (LEAP™). Cells cultured in 96-well plates are selectively ablated in a perfectly circular area with a diameter of 25% of the well diameter. Again, an imprecise control of the laser can result in heat build-up that burns the surrounding cells in an indeterminate manner. Moreover, to perform damages with these sophisticated optical procedures it is necessary to make the use of instruments not available to all laboratories.

Despite the advantages and ease of use for studying cell migration and proliferation, which are essential for understanding the processes of angiogenesis [51] and re-epithelialization [52], in vitro 2D assays are not able to recapitulate the complex conditions of a wound. First, these kinds of experimental procedures usually involve only one cell line. In-depth studies of wound healing have shown that it proceeds in a well-organized and controlled way towards an accurate restoration of three-dimensional architecture when different cell types work together and interact, especially cells of the immune system that control the entire process from the beginning to the end. But, most important, 2D assays do not recapitulate the exact microenvironment of a wound since they lack the extracellular scaffolding matrix (ECM), that establishes intercellular signals or networks that direct cells to grow, migrate, and differentiate.

## 4. Full-Thickness Models: Toward the Replication of Deep Wounds Repair

The skin is the most extensive tissue exposed to the external environment, which means that it is vulnerable and more susceptible to injury and trauma. For this reason, the integumentary system is often the one most reproduced in vitro to analyze its wound healing dynamics. Based on the previously described methods, the absence of a dermis featuring the 2D models, means that neither the ECM remodeling, nor the dermis-epidermis cross talk have been considered during the in vitro studies of the wound. Bilayer skin models comprising dermis and epidermis have been developed to increase the mimicking potential of the wound healing process in vivo [53,54,55,56,57,58]. The tissue engineering approach, aiming at reproducing a functional and structural dermal substitute, relies on the creation of three-dimensional scaffolds as an ECM analogue to guide cell adhesion, growth and differentiation. Regardless of their origin, synthetic or natural, the scaffolds used to replicate the human dermis can be divided into two main categories: exogenous or endogenous. In an exogenous dermis a pre-existing scaffold is used to accommodate human fibroblasts. Synthetic [57] and natural biopolymers [53], as well as human decellularized dermis [54,55,59] fall into this approach. On the contrary, if the fabrication process of the dermis is based on established bioengineering processes able to induce human fibroblasts in synthesizing and assembling their own ECM, the resulting dermis is considered as an endogenous engineered dermis. In this category fall engineered dermis is obtained by both cell-sheet engineering [60] and dermal microtissue assembly approaches [58,61,62].

### 4.1. Deep Wound Models Based on Exogenous Dermis Equivalents

**Figure 3:**
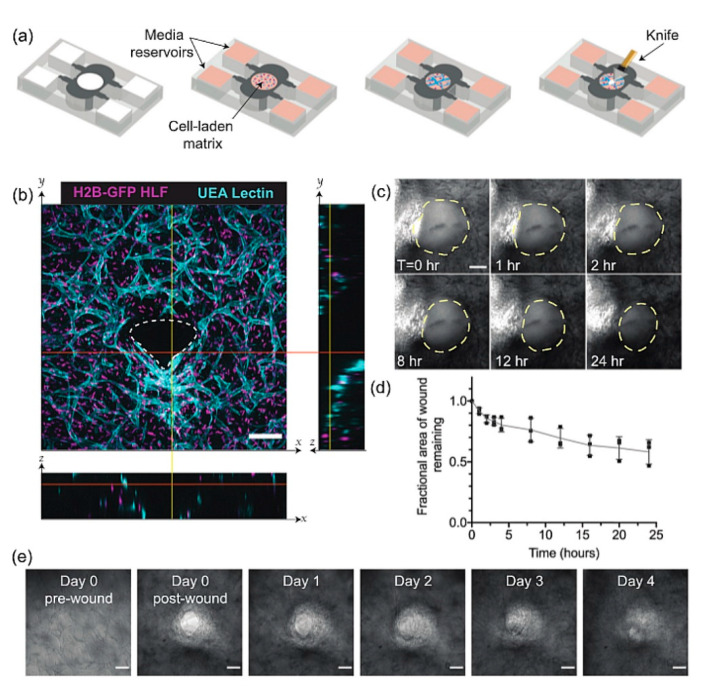
Damaged 3D exogenous dermis equivalent: (**a**) Schematic representation of the experimental design: endothelial cells and fibroblasts embedded in a fibrin gel are inserted in a PDMS device. The 3D vascularized tissue is allowed to mature for 3 days before cutting with a diamond knife, (**b**) Immunofluorescence image of the wounded vascularized tissue one day after cutting. Z-projection, both in x-z and y-z coordinated, indicates the wound depth and white dotted lines delineate wound boundaries, (**c**) 24 h-brightfield time-lapse acquisitions with wound borders highlighted by yellow dotted lines, (**d**) Quantification of wound area reduction over 1 day, (**e**) Brightfield images of the tissue up to wound closure day (day4). Reproduced under the terms of the CC BY 4.0 license from Tefft JB, Chen CS, Eyckmans J. Reconstituting the dynamics of endothelial cells and fibroblasts in wound closure, 2021;016102. [71] Copyright © 2021, The Author(s); published by AIP Publishing.

Exogenous scaffolds [63] have been used to build full thickness models subjected to different wounding methods: mechanical, thermal, or laser-induced damage (Table 3) [64,65,66]. Early works by Falanga et al. [67] reported the use of bilayer skin models, with the dermal component made of reconstructed rat tail collagen containing human fibroblasts [67]. After a mechanical injury was obtained by using a skin mesher, the wounded region was investigated in vitro for up to 6 days in terms of keratinocytes migration, proliferation, re-epithelization, and inflammatory response. During the culture period, it was observed a complete re-epithelization and closure of the defect; in addition, the expression of acute proinflammatory cytokines was detected with a peak 24 h post-injury. Other groups have used human decellularized dermis (DED) populated with human fibroblasts [68,69]. These models have been used to study the effect of both biopolymers (i.e., fibrin) and growth factors on wound closure. Geer et al. [68], used a full thickness model consisting of DED and keratinocytes to investigate the role of fibrin in the closure of partial wounds [68], highlighting its beneficial role in promoting the re-epithelization process. In another work by Yan et al. [69], a bilayer skin model based on DED, was injured with a biopsy punch, creating a 4 mm wound that extended through both epidermis and dermis. The wounded area was filled with a biomimetic hydrogel to mimic the formation of the fibrin clot; furthermore, re-epithelization and dermis closure were studied by using histological and immunostaining techniques. Although these systems represent an advance in the field, compared with 2D approaches, the authors recognized crucial limitations of the models, such as the lack of immune response and the absence of other cell types (e.g., melanocytes, Langerhans’ cells, microvascular endothelial cells). Moreover, the importance of reliable and robust 3D wound healing models has also pushed the development of standardized wound healing models by using 3D printing technology as well as computer-assisted mechanical wounds [60]. Nevertheless, most models that replicate the full-thickness wound healing process, are used as in vitro platforms to screen molecules able to speed up the re-epithelization step, by using the dermis as a supporting 3D matrix for the epidermis. The presence of the dermis should allow the study of more complex phenomena underlying skin regeneration, such as the formation of the fibrotic scar. Indeed, wound closure is not just a cellular phenomenon: together with inflammation and immune response, it is necessary to consider the complex machinery involving different cell types, ECM remodeling, and cell-ECM crosstalk. In this direction, several studies have been performed to investigate the interactions between fibroblasts, immune cells, and endothelial cells and the force distribution and ECM remodeling during the closure of deep wounds (Figure 3) [70,71,72,73,74].

A wound on-chip model developed by Biglari et al. [70], comprises three communicating compartments hosting fibroblasts, endothelial cells (EC), and M1/M2 macrophages respectively seeded in either rat tail collagen or Matrigel biopolymers. The system was specifically designed in order to replicate the step occurring just after the hemostasis phase, known as the inflammation phase. In this work, M1 macrophages released different cytokines that stimulate the migration of fibroblasts and endothelial cells (ECs). After the transition of M1 to M2 macrophages (an anti-inflammatory cell type), key cytokines, such as TNF-α, were produced to induce the differentiation of fibroblasts into myofibroblasts, ECM production, and angiogenesis. By using this device, the pro- versus anti-inflammatory effects of M1 and M2 macrophages were assessed. To validate the system, an anti-inflammatory compound, Dexamethasone, was used as a proof of concept to demonstrate and validate the feasibility of using this wound-on- chip device to examine the impact of active compounds on inflammation. Although this experimental model provided useful information on the role of M2 on fibroblast migration and differentiation, neither mechanistic aspects nor information on the remodeling of neo-ECM was reported. More detailed investigations reporting mechanistic studies and ECM remodeling have been obtained by inducing a cut in rat tail collagen gels containing either fibroblasts alone [73] of fibroblast and endothelial cells [71]. Sakar et al. [73], developed an optically accessible device hosting rat tail collagen microtissues populated with 3T3 fibroblasts injured by an incision to simulate a deep wound occurring in the dermis. The study was conducted in the absence of the epidermis layer since the attention was posed on the mechanistic aspects that occur during wound closure at the dermis level. Tensile force microscopy, time-lapse imaging, and immunostaining revealed that fibroblasts closed the gap by means of a coordinated action of force-dependent contraction involving the entire tissue. Also, circumferential fibroblast migration around the edges of the wound and a concomitant assembling of fibronectin around the wound edges was observed as the mechanism by which the cells restored tissue integrity. The advancement of this model was proposed by Tefft et al. [71], in which the engineered dermis was populated by a co-culture of fibroblasts and endothelial cells. After cutting, the authors found remarkable differences between their 3D model and 2D wound healing model. Indeed, while the latter showed fast migration of endothelial cells after the wound, the dynamic in the 3D environment was quite different. It was observed that fibroblasts were engaged in the closure and in the deposition of new ECM components (i.e., fibronectin and collagen type III); the vessel structures, on the other hand, were primarily restricted to the wound periphery, and movement of the vascular structures was associated with tissue contraction of the initial matrix, adding a new perspective to the understanding of blood vessel dynamics in early stages of healing. In this context, tissue engineering, using cells growing within 3D supports, reduces the gap between flat cell cultures and physiological tissues and preserves the differentiated tissue-specific phenotype. Although 3D exogenous matrices do not fully mimic the native context, they are valuable tools to conduct fast studies on mechanistic phenomena occurring during the closure process.

### 4.2. Deep Wound Models Based on Endogenous Dermis Equivalents

**Figure 4:**
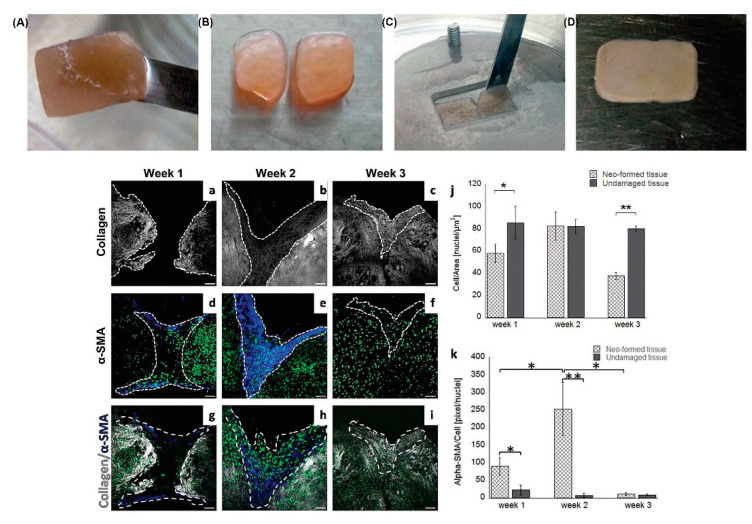
Damaged 3D endogenous dermis equivalent: (**A**–**D**) Workflow of the 3D-HDE (human dermal equivalent) cutting. Specifically, after 4 weeks of maturation, a 3D-HDE is cut with a scalpel into two parts; then, these parts are reallocated close together in a maturation chamber to reproduce a first intention wound healing in a dynamic regime. (**a**–**c**) SHG of the 3D-HDE after wounding over 3 weeks illustrates the neoformation of collagen fibers, (**d**–**f**) Qualitative evaluation of an α-SMA expression, over 3 weeks from wounding, shows how this protein is abundant during the second week which approximately corresponds to the proliferation phase, as the in vivo counterpart. This expression is suppressed during the third week which is reasonable since remodelling phase is starting, (**g**–**i**) Merge of the signals previously described, (**j**,**k**) Quantitative analyses of the number of cells per area and α-SMA production per number of cells. Symbols “*” and “**” stay for significant differences with *p* < 0.05. Reprinted from Lombardi B, Casale C, Imparato G, et al. Spatiotemporal Evolution of the Wound Repairing Process in a 3D Human Dermis Equivalent. Adv Healthc Mater 2017; 6:1–11 [79] with permission from John Wiley and Sons.

As discussed so far, the dynamics involving the dermis during the closure process, play a crucial role in understanding the phenomena underlying the closure of deep wounds. It is well accepted that the inflammatory process and the pro-inflammatory agents released during this step, strongly affect the neo-ECM remodeling leading to the formation of the fibrotic scar. In this perspective an ideal engineered dermis should be able to replicate, not only the cellular events, but also the complex ECM dynamics involved during the morphogenesis of the dermis: the synthesis and 3D assembling of the neo-ECM components (e.g., collagen, elastin, hyaluronic acid, and fibronectin). In this context, the exogenous matrices provide partial information about the ECM dynamics. Although interesting phenomena such as cell differentiation, migration, and mechanical signaling can be studied, different limitations appear. For instance, from a mechanical point of view, the 3D context generated by the exogenous matrices is quite different in terms of local stiffness and hydration from the native one. Also, exogenous matrices do not contain all the ECM components that are able to provide the native regulatory context useful for the correct maintenance of fibroblasts. The lack of both correct mechanical and compositional microenvironment makes the exogenous matrices an oversimplified 3D context, as the fibroblast receptors are not properly engaged in physiological interactions with their own ECM. This can result in aberrant responses. Moreover, exogenous matrices are unable to modify their composition/architecture during the progression of a pathological status as the native ECM does [76]. In fact, compared to the exogenous matrices, the model based on the use of an endogenous ECM has revealed the ability to replicate in vitro crucial phenomena related to the ECM functionalities: (i) morphogenesis, neo-synthesis, assembly, and ECM turnover [61], (ii) modification of ECM composition/architecture during a pathological state [75,77,78], (iii) correct dermis/epidermis crosstalk via the spontaneous formation of both rete ridge profile and hair follicle-like structures [58]. Lombardi et al. [79], used an endogenous 3D human dermis equivalent obtained by inducing fibroblasts to synthesize and assemble their own ECM (Figure 4). The dermis model was surprisingly indistinguishable from the native one in terms of composition, architecture, and mechanical properties, being collagen, elastin, fibronectin, and hyaluronic acid correctly assembled in the extracellular space during the formation process. Once the dermis was correctly assembled, the engineered tissue was cut, and the closure was observed for up to 4 weeks. During the closure steps, in addition to observing cellular events such as fibroblasts invasion and differentiation, relevant ECM dynamics were also detected. A provisional matrix of fibronectin and hyaluronic acid was deposited in the gap in the first week, followed by the deposition and assembly of a collagen matrix in the successive weeks. Furthermore, the collagen network assembled in a scar-like architecture that showed an orientation of the collagen fibers orthogonal to the cut direction. Finally, the composition of the neo formed ECM differed from that of the undamaged tissue. Overall, the spatiotemporal cellular and extracellular events occurring during the closure resembled those that occur during the formation of scar tissue in the native dermis. These data suggested that, even in absence of an acute inflammatory response, adult fibroblasts are involved in a repair process instead of a regenerative one. Moreover, the use of endogenous engineered dermis allows in vivo-like studies after the application of different damaging sources which share with the mechanical wounds the remodeling of the ECM. For instance, in a study by Casale et al. [78], a human skin equivalent (HSE) was presented, consisting of a dermis made up entirely of fibroblasts embedded in their own extracellular matrix on which epidermal cells were seeded. HSEs were subjected to UVA rays exposure and the results obtained showed that, as a result of damage, these skin equivalents were not only able to replicate the complex cells-ECM interactions but provided evidences of the reorganization of the collagen network after the photodamage.

## 5. The Need of Immune Response In Vitro

**Figure 5:**
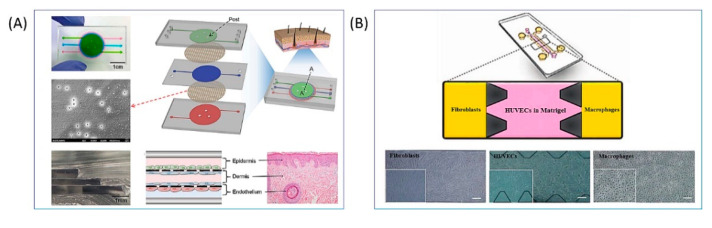
Skin inflammation on chip: (**A**) Representation of an inflamed skin-on-chip configuration composed of a three-layers PDMS device and two PET porous membranes. Each layer corresponds to a cell line comprising human skin and, in particular, from bottom to top, endothelial cells, fibroblasts, and keratinocytes. Reproduced under the terms of the CC BY 4.0 license from Wufuer M, Lee GH, Hur W, et al. Skin-on-a-chip model simulating inflammation, edema and drug-based treatment. Sci Rep 2016; 6:1–12. [74] Copyright © 2016, The Author(s); published by Springer Nature. (**B**) Simulation of inflammation triggered by a wound through a microfluidic device. The microfluidic chip consists of three layers that host the cells that directly participate in this physiological process. Fibroblasts and macrophages are fluxed through the lateral channels while, in the central one, is added a Matrigel, which reproduces ECM, together with endothelial cells. Reprinted from Biglari S, Le TYL, Tan RP et al. Simulating Inflammation in a Wound Microenvironment Using a Dermal Wound-on-a-Chip Model. Adv Healthc Mater 2019; 8:1801307 [70] with permission from John Wiley and Sons.

The epilogue of the wound healing process is the simple reconstruction of the compromised tissue architecture. The event may end with chronic, hard-to-heal wounds or tissue fibrosis, both of which represent an enormous burden on the health system worldwide and are associated with significant patient morbidity and mortality. Consequently, it is ever more pressing the request to acquire more information about the mechanisms that regulate the wound restorative progression and that lead to the identification of new treatments which aim to improve the injuries treatment. It is known that damage repair requires a complex and delicate interaction between the immune system, keratinocytes, and dermal cells which provide signals that guide all four normal stages of wound healing. The use of animals for preclinical research has been the standard method for decades, contributing to important advances in the understanding of human biology [80]. However, because wounds do not always heal rapidly due to different etiologies, no animal model can be considered universally valid while a specific configuration could mimic a desired aspect of the human condition and provide a complete understanding of this intricate mechanism. To meet the requirements regarding the species-specific and the cause of the wound, ex vivo skin derived from skin explants was proposed as a viable model, which made it possible to assess wound healing in human tissues, as it has all the native structures of human skin. However, due to its limited availability and life cycle and together with the lack of immune cells and blood supply, its use is restricted to very limited applications [81]. Since the adoption of the principles of the 3R and the animal testing ban [82], the development and use of alternative in vitro platforms have increased significantly. Preclinical screening of new substances for better healing can be evaluated with 2D tests and 3D models of more complex skin wounds, as previously described. In this digression on useful and valid models for detailed assessment of healing dynamics, 3D models of skin seem to be the most appropriate as they ensure the implementation of a more physiologically relevant environment. Full-thickness skin models, consisting of a dermal compartment with fibroblasts and a fully differentiated epidermis on top of it, better recapitulate what happens in vivo. Indeed, the paracrine signaling among residing cells is ensured and the fibroblast’s influence on re-epithelialization is reproduced with good accuracy. However, the use of fibroblasts and keratinocytes alone is a limitation to the progress of skin models as validated preclinical platforms for wound healing investigation. This co-culture reproduces only a fraction of how the skin appears when a chronic wound occurs. The lack of accessory structures such as the vasculature or an immune system limits the use of skin models to study more complex processes [83]. The preclinical development phase of a drug would gain greater impact and significance by the use of in vitro models able to physiologically recapitulate the native counterpart. To improve the in vitro models already available and implemented for the study of lesions on the skin, the missing dowel is the immune system responsible for triggering the inflammatory response as it has already been tried to do with the previously described microfluidic devices (Figure 5).

**Figure 6:**
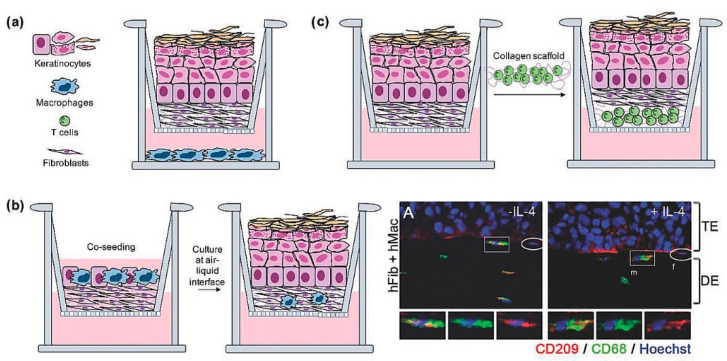
Immunocompetent skin models: (**a**–**c**) Schematic representation of macrophages or T cells co-seeded with a full-thickness skin equivalent. Reprinted from Pupovac A, Senturk B, Griffoni C, et al. Toward Immunocompetent 3D Skin Models. Adv Healthc Mater 2018; 7:1–11 with permission from John Wiley and Sons. (**b-right panel**) Experimental results of pan macrophages and M2 macrophages migration towards the epithelium. Reproduced under the terms of the CC BY 3.0 license from Linde N, Gutschalk CM, Hoffmann C, et al. Integrating macrophages into organotypic co-cultures: A 3D in vitro model to study tumor-associated macrophages. PLoS One 2012; 7. [84] Copyright © 2012, The Author(s); published by Plos.

There is a need to recreate an organotypic equivalent of immunocompetent skin to be damaged and in which all the steps leading to a chronic wound or fibrotic tissue can be traced. At present, several immunocompetent skin models have been developed for a detailed study of specific diseases (Figure 6).

The incorporation of melanocytes has made it possible to study the skin’s response to UV radiation [85] and the progression of melanoma [86], Langerhans cells have been successfully implemented to study their recruitment to the epidermis for skin sensitization analysis [87,88], the co-culture of dendritic cells in skin equivalents has allowed the assessment of their role in cases of allergic contact dermatitis [89] and the introduction of T cells in these models has allowed an in-depth study of *Candida Albicans* infection [90]. When studying wound healing, of all the cellular components involved in the process, macrophages and T cells are the only cell types that persist in the wound area at all stages. Whether they reside on the skin or are recruited by precursors circulating in the blood, their contribution to healing is crucial and a dysfunction of their activity results in an ineffective resolution of inflammation. Therefore, the incorporation of these cells is of utmost importance and relevance for the exploration of new healing therapies. During the healing of wounds, both macrophages and T cells adopt a dual phenotype as a result of microenvironmental signals, each contributing to the process with distinct functions. The dual role and function of these innate and adaptive immune system cells is a challenge when it comes to implementing them in 3D skin models. Initial attempts to include T cells for an in vitro lesioned skin model were done by Lorthois et al. [91] and Shin et al. [92]. In these works, it was shown that starting from both lesional psoriatic cells [91] and skin cells derived from healthy individuals [92], the infiltration of T cells into these 3D skin equivalent constructs was validated by anti-CD3 immunofluorescence staining. Furthermore, these immunocompetent skin models can secrete more pro-inflammatory mediators than 3D skin without immune cells under the same experimental conditions. Linde et al. [84] and Bechetoille et al. [93] tried to include macrophages into in vitro skin cultures. In the former work, the group co-seeded macrophages with a tumor epidermis stimulating the overall model with IL-4. In the latter, the authors co-cultured macrophages with fibroblasts and then, stimulated this construct with lipopolysaccharide to trigger an inflammatory response. In both studies, cell survival has been proved for 1 week [93] and 3 weeks [84] but in one case the epidermis was absent and in the other, the presence of a diseased epidermis limited the relevance of the models. It is, therefore, necessary to incorporate macrophages or T cells into healthy engineered skin models to subsequently reproduce the effect of mechanical or chemical trauma. This would significantly and remarkably increase the relevance of wound healing investigations. An effective method of triple co-culture should be found, in which at least dermal fibroblasts, keratinocytes, and macrophages or T cells are cultured together, to obtain potentially improved skin equivalents needed for the evaluation of new impaired healing therapies aimed at increasing the effect of preclinical models on the patient. Working in this direction, these immune-competent endogenous models may be perfect emulators of the human trauma response. For instance, they could be used to validate on human equivalents the use of non-thermal plasma treatments, which so far was only tested on murine models [94,95]. These treatments have not only been shown to speed up the repair of wounds but have also had a considerable anti-fibrotic role on the reduction of TGF-β1 released by macrophages and on the subsequent expression of αSMA and collagen I deposition.

## 6. Conclusions

In this work, the in vitro wound healing models was reviewed in the light of their effectiveness in replicating the complex machinery leading to the formation of the fibrotic scar. The possibility to predict in vitro the effects of specific compounds, or treatments, on the evolution of a deep skin wound can lead to huge improvements in the prevention of disabling scars. Nevertheless, by analyzing the literature, it must be recognized that we are still far from the in vitro recapitulation of the entire process. Two-dimensional models, composed of only keratinocytes allow to study of superficial lesions, providing information on cell motility and on the capability of molecules in speeding up the re-epithelization process [35,40,41,44,46] but they do not provide any improvement in the understanding of the formation of severe scars. With the aim to develop a more reliable 3D full-thickness system, skin models have been developed which consist of both ex vivo explants and engineered skin models. While the former are poorly used due to their in vitro limited viability, the latter have gained more attention. Engineered skin models comprise a dermis compartment populated with fibroblasts and a differentiated epithelium. It has been shown that after a mechanical wound, the presence of the dermis allowed the replication of phenomena that cannot be observed in 2D models at all, dynamics implicated in the formation of a provisional matrix, and the mechanical interactions among fibroblasts and the ECM [71,73]. However, dermis models, composed of exogenous scaffolding materials, fail in replicating the key events that lead to the formation of the fibrotic scar: the impaired synthesis and assembling of the ECM. In this regard, endogenous-based tissue-engineered dermis appears more promising as fibroblasts, other than differentiating towards myofibroblast phenotype, can re-synthesize, and assemble new ECM components in the wounded area replicating the key events involved in the fibrotic tissue formation. Such models have been shown to possess the unparallel capability to replicate the pathological events occurring in the native dermis after different wound inductions (mechanical and UV radiations) due to their capability to undergo impaired ECM remodeling. Conversely, the exogenous scaffolds are insensitive to the same exogenous stimuli [58,75,79]. This endogenous dermis seems the more promising 3D models and can be further complicated by introducing other cell populations such as specific immune cells [87,89,90,93]. This will allow to recreate the complex interplay exiting among inflammation, differentiation, and ECM remodeling providing a new tool to deeply understand the mechanisms involved in the scar formation with the final aim to aid the development of compounds and therapeutic approaches promoting scar-free healing. Besides mechanical damages, the ECM remodeling and inflammation are the response of the human skin to other factors also. Therefore, the proposed 3D models can increase our knowledge of skin remodeling during aging and/or after the application of extrinsic stressors (i.e., foods, UV, smoke, pollutants, drugs, lifestyle). The knowledge of such mechanisms can help the development of new drugs and treatments to preserve skin functions, paving the way to new frontiers in the field of wellness.

## Figures and Tables

**Table 1:** Main cells and molecules involved in the wound healing process.

Cell Type	Activated by	Molecules Released	Effects
PLATELETS [21]	Exposure to the underlying collagen and vWF after blood vessel rupture	PDGF, TGF-β, bFGF, KGF, EGF, IGF	Fibrin clot (scab) formation; enhance neutrophils, macrophages, fibroblasts, and endothelial cells chemotaxis and infiltration
ENDOTHELIAL CELLS (HEMOSTASIS PHASE) [21]	Blood vessel injury	Prostaglandins, Leukotrienes	Vasodilation and platelets disassembly; increase in vascular permeability and in leukocytes chemotaxis and adhesion
ENDOTHELIAL CELLS (PROLIFERATION PHASE) [21]	Tissue hypoxia, bFGF, KGF, VEGF, TNF-α, TGF-β, thrombin	Proteolytic enzymes, matrix MMP	Angiogenesis
DERMAL MAST CELLS [21]	Complement system (C3a and C5a), physical stimuli (heat or mechanical injury)	Histamine, TNF-α, IL-1, TGF-β, PDGF, serine protease, chymase, tryptase, Prostaglandins, Leukotrienes	More leaky and permeable blood vessels; breakdown of the ECM to pave the way for fibroblasts and endothelial cells proliferation
NEUTROPHILS [23]	Factor released by platelets, by-products of bacterial degradation	ROS, NO, antimicrobial peptides, antimicrobial proteases, IL-17, VEGF	Phagocytosis; antimicrobial function; wound debridement
MACROPHAGES (M1 PHENOTYPE—PRO- INFLAMMATORY) [23]	Derived from chemotaxis of migrating monocytes activated by bacterial products, complement degradation products (C5a), and factor released by platelets and neutrophils	Proteinases, antimicrobial peptides and proteases, TNF-α, TGF-β, IL-1, IL-8	Phagocytosis; antimicrobial function; wound debridement
MACROPHAGES (M2 PHENOTYPE—ANTI- INFLAMMATORY) [23]		Proteinases, TGF-β, EGF, PDGF, TNF-α, IL-1, IFN-γ, IGF, IL-6, Fibronectin, bFGF, VEGF	Matrix synthesis regulation; cell recruitment and activation; angiogenesis
T LYMPHOCYTES [26]	IFN-γ released by macrophages	IL-2, IFN-γ, IL-4, IL-10, TGF-β, TNF-α, FGF	Macrophages production and differentiation regulation; synthesis and proliferation of fibroblast
EPITHELIAL CELLS/ KERATINOCYTES [21]	Mainly, EGF, secreted by platelets, and TGF-α produced by macrophages, platelets, and keratinocytes	bFGF, VEGF, TNF-α	Re-epithelization
FIBROBLASTS/ MYOFIBROBLASTS [21]	PDGF, TGF-β, FGF, EGF, and IGF released by platelets and macrophages	Collagen type I and III, elastin, GAGs, adhesive glycoproteins	Matrix components synthesis; wound contraction

Abbreviations: Insulin-like growth factor (IGF), ROS (Reactive Oxygen Species), NO (Nitric ox-ide), glycosaminoglycans (GAGs), Platelet Derived Growth Factor (PDGF), Transforming Growth Factor (TGF), Keratinocyte Growth Factor (KGF), Epidermal Growth Factor (EGF), Fibroblast Growth Factor (FGF), Metalloproteinases (MMP), Tumor Necrosis Factor (TNF), Interleukin (IL), Vascular Endothelial Growth Factor (VEGF), Interferon (IFN), von Willebrand factor (vWF).

**Table 2:** Common 2D models used for bidimensional wound healing assays.

2D Models	Aim	Ref.
Keratinocytes monolayers	Study of the migration behavior to reproduce re-epithelialization process	[33,38,39,42,44]
Fibroblasts monolayers	Evaluation of the migratory potential to study their speed, persistence, and polarity during granulation tissue formation	[31,32,35,46]
Endothelial cells	How endothelial cells migrate and grow towards an angiogenic stimulus to form sprouts	[43,45,48,50]
**2D wound assay**	**Description**	**Ref.**
Scratch Assay	Scratch ofa confluent monolayer of cells with pipette tip, cell scrapers, toothpicks or metallic micro indenters	[30,31,32,33,35,36,37,41,42,43,44,50,51]
Stamp Wound Assay	Determine a lesion on the cell culture with a high-pressure force	[38,39]
Thermal injury Assay	Injury a specific zone of the cell monolayer with very high or low temperatures	[45]
Electrical Injury Assay	Destruction of a cell portion by applying an electric current	[46,47,48]
Optical Injury Assay	Formation of a wounded area by means of a laser beam	[49]

**Table 3:** Common 3D models used for tridimensional wound healing assays.

3D Models	Aim	Ref.
**Exogenous skin wound models**		
Fibroblasts-populated Rat tail Collagen I + keratinocytes	Besides new tissue formation, these in vitro platforms enable us to screen molecules able to speed up the re-epithelization step	[62,65]
Fibroblasts in DED + keratinocytes		[64,66,67]
**Exogenous dermal wound models**		
Fibroblasts-populated Rat tail Collagen I	Reproduction of the complex processes concerning ECM remodelling and cell-ECM crosstalk	[71]
Fibroblast and endothelial cells embedded in Rat tail Collagen I and fibrinogen		[69]
**Endogenous dermal wound model**		
Fibroblasts embedded in their own ECM	Able to replicate in vitro morphogenesis, neo-synthesis, assembly, ECM turnover, and modification of ECM composition/architecture during a pathological state.	[75]
**3D wound assay**	**Description**	**Ref.**
Mechanical Injury	Creation of a wound through a scalpel, a biopsy punch, or a rotating drill	[66]
Laser wound	Tissue ablation in a specific area by a laser beam	[62]
Thermal injury Assay	Deep burn wounds generated through the contact with a stainless-steel rod connected to a soldering iron	[64]
